# The Phonendoscope

**Published:** 1898-02

**Authors:** 


THE PHONENDOSCOPE.—This instrument, destined to replace the stethoscope, was devised by Drs. Aurelio Bianchi, of Rome, and Eugenia Bazzi, of Florence, Italy.

It consists of three principal parts: The resonator; a detachable hard-rubber diaphragm, and a metallic rod with flattened end which screws into the detachable diaphragm.

The resonator is the essential part, and consists of a plano-concave metallic disk, $2\frac{1}{4}$ inches in diameter and $\frac{3}{4}$ inches thick, the concave side of which is closed by means of a thin, hard-rubber diaphragm, similar to the receiver (or transmitter) in a telephone, but with a slight bulge in the centre similar to the flexible bottom of an oil-can. Two soft-rubber tubes tipped with metal are inserted into holes in the plane side of the resonator; the other end of the tubes having self-retaining ear-pieces.

The advantages of the phonendoscope are that it enables us to appreciate more clearly the normal and pathologic sounds emitted by the organs of the body; many acoustic phenomena which are not audible by ordinary means of auscultation are rendered clear and distinct; it is used to ascertain the form, position, and thickness of separate viscera, replacing percussion; it does not present the disadvantages of all other stethoscopes, of limiting the area of auscultation to an exceedingly small area of the lung; but pulmonary sounds are gathered from a considerable distance and from a considerable depth, and in many cases the cardiac sounds and murmurs are heard with unusual distinctness and clearness; in myocarditis, when the cardiac sounds cannot be distinguished with the stethoscope, they can be distinctly heard with the phonendoscope, even through several thicknesses of clothing; the phonendoscope is as great an improvement over the stethoscope as the latter was over immediate auscultation, and we have in it a most perfect aid to accurate diagnosis.

